# Recurrent neck myxofibrosarcoma: a case report 

**DOI:** 10.1186/s13256-021-03053-4

**Published:** 2021-10-01

**Authors:** Chiara Pagnoni, Luca Improta, Rossana Alloni, Francesco Mallozzi Santa Maria, Irene Aprile, Beniamino Brunetti, Carlo Greco, Bruno Vincenzi, Alessandro Gronchi, Sergio Valeri

**Affiliations:** 1 Campus Bio-Medico University of Rome, Via Alvaro del Portillo, 200 - 00128 Roma, RM Italy; 2 IRCCS Fondazione Don Carlo Gnocchi-Onlus, Florence, Italy; 3 Fondazione IRCCS Istituto Nazionale dei Tumori, Via Venezian, 1, 20133 Milan, MI Italy

**Keywords:** Soft tissue sarcoma (STS), Sarcoma referral centers (SRCs), Myxofibrosarcoma (MFS), Preoperative radiotherapy, Wide surgical margins

## Abstract

**Background:**

Myxofibrosarcoma (MFS) is a rare soft tissue sarcoma with a high recurrence rate and a low risk of distant metastasis. It occurs mainly in the extremities of elderly men. Head and neck MFS is extremely rare. Surgery is the cornerstone of treatment. The role of radiotherapy (RT) and chemotherapy (CHT) on MFS is still debated.

**Case presentation:**

A 67-year-old Caucasian man presented to our sarcoma referral center (SRC) with a history of MFS of the neck excised with microscopic positive surgical margins in a non-referral center. Staging imaging exams did not reveal distant metastasis. After a multidisciplinary discussion, preoperative RT was administered with a total dose of 50 Gy followed by wide surgical excision. Histological examination was negative for viable tumor cells. No relapse occurred during the 24-month postoperative follow-up.

**Conclusions:**

The case described suggests the importance of planned combined treatments with both RT and surgery for high-grade soft tissue sarcoma. RT seems to be promising within this specific histotype. Close follow-up is advisable in all cases. Further studies are needed to confirm if the observed efficacy of combined treatments results in a prolonged time of disease-free survival and overall survival.

## Background

Myxofibrosarcoma (MFS) is one of the most common types of soft tissue sarcoma (STS) in adult patients [1], representing approximately 5–10% of all STS [2]. The World Health Organization (WHO) defines MFS as a malignant connective tissue neoplasia of fibroblastic origin [2]. It mainly presents as a painless, slowly growing mass in the lower and upper extremities or the superficial trunk [3, 4]. MFS typically affects patients between the sixth and seventh decades of life [1], and men to a larger extent than women [2].

Head and neck MFS is extremely rare, with a reported rate of about 3–10% [2]. Its rarity is a potential source of diagnostic difficulty. In fact, for tumors present on the head and the neck, the histological differential diagnosis includes atypical fibroxanthoma, pleomorphic dermal sarcoma, myxoid spindle cell squamous cell carcinoma, myxoid dermatofibrosarcoma protuberans, spindle cell melanoma, and myxoid fat-free spindle cell lipomas [5]. Recognition of the characteristic histological features of MFS in conjunction with judicial use of immunohistochemical stains allows for accurate diagnosis [5]. Therefore, patient evaluation in sarcoma referral centers (SRCs) is critical for ensuring appropriate pathological diagnosis and management.

Local recurrence is the main risk in patients with MFS. For this reason, according to European Society for Medical Oncology–European Reference Network on Rare Adult Cancers (ESMO-EURACAN) Clinical Practice Guidelines [6], wide surgical excision combined with radiotherapy (RT) provides the best chance for enhancing local control in this condition [7]. However, the need for wide surgical excision in such a complex anatomical district may compromise the functional outcome of these patients. For that reason, the presence of a dedicated physiotherapist to provide a specific motor recovery program is essential. No data about the motor performance of sarcoma patients after surgery and the eventual results of rehabilitation are reported in literature.

We illustrate the case of a recurrent MFS arising in the left trapezius muscle, treated with RT followed by wide surgical excision, showing a complete pathological response to preoperative treatment.

We also report the patient’s functional recovery after the rehabilitation program.

## Case presentation

A 67-year-old Caucasian man presented to our referral center in January 2018 with a history of recurrent MFS of the neck. One year earlier, the patient had undergone inappropriate surgery, without preoperative core-needle biopsy, with microscopic positive surgical margins (R1 resection), in a non-referral center. The histological examination diagnosed a pleomorphic sarcoma, showing 30% of tumor cells positive for Ki-67. No postoperative treatments were provided.

**Fig. 1:**
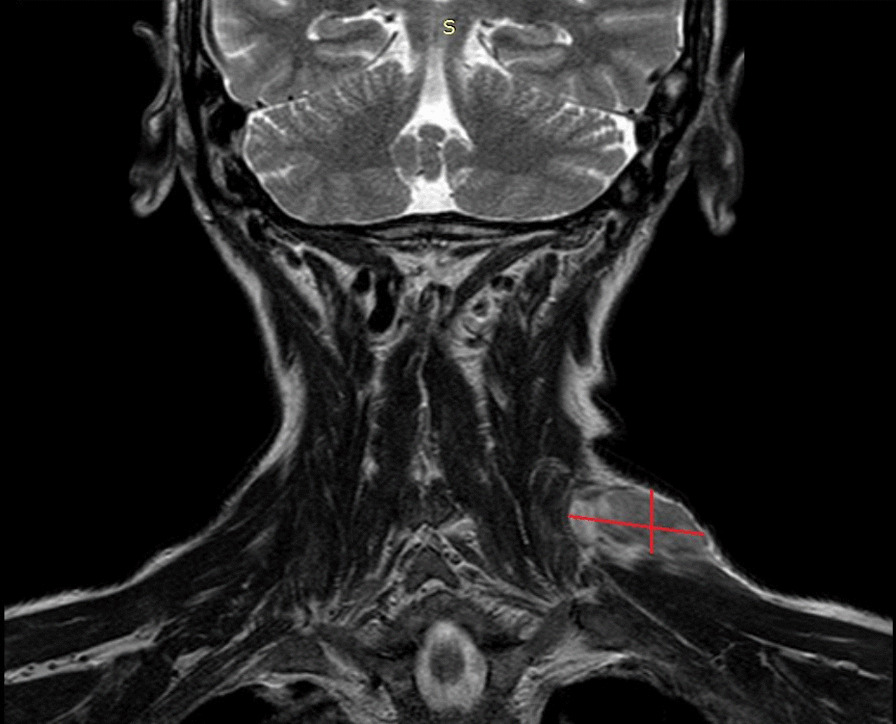
Magnetic resonance imaging at diagnosis of recurrence, showing a mass measuring 50 × 20 mm (diameters shown by red lines)

At our first medical examination, a stiff, immobile, and painless 5-cm mass was observed on the left posterior neck. Neck magnetic resonance imaging (MRI) showed a 50 × 20 mm mass with initial invasion of the left paravertebral muscle (Fig. 1). A positron-emission tomography/computed tomography (PET/CT) scan did not reveal distant metastasis. A pathological re-examination of the histological specimen was performed, and a pathological reclassification as MFS was made.

The case was discussed in our multidisciplinary tumor board (MTB), where the decision was made for preoperative RT, with a total dose of 50 Gy (fractionated into 200 centigray/daily).

**Fig. 2:**
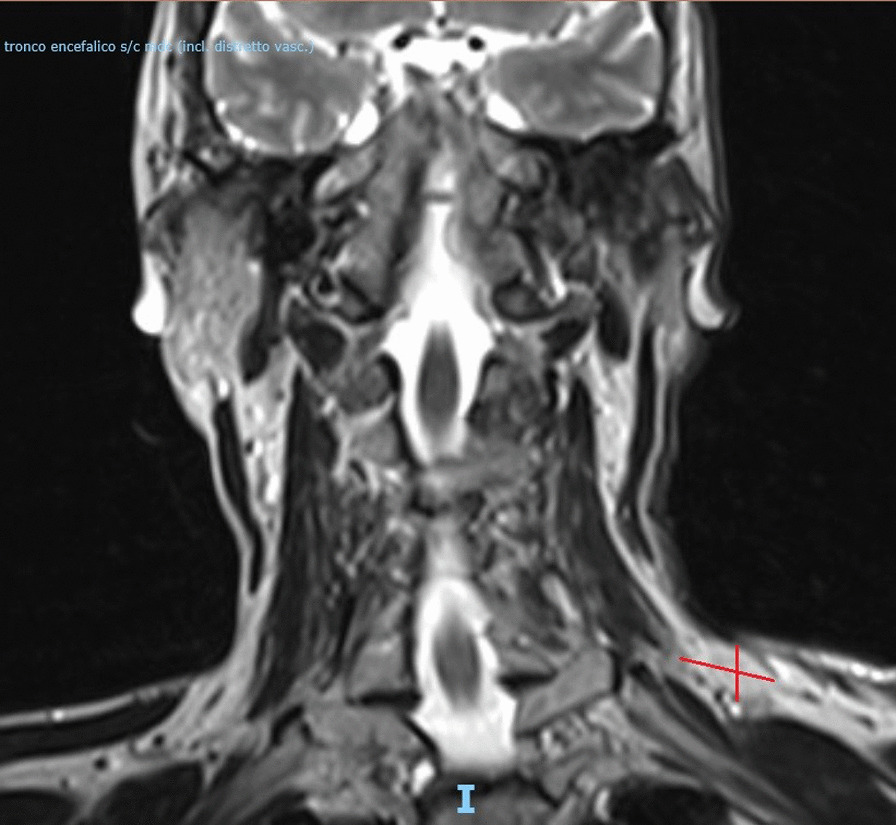
Magnetic resonance imaging after 1 month of radiotherapy, showing a dimensional decrease of the mass (22 × 15 mm) (diameters shown by red lines)

Post-RT MRI performed 1 month later showed a partial radiological response to treatment, with a mass measuring 22 × 15 mm (Fig. 2).

**Fig. 3:**
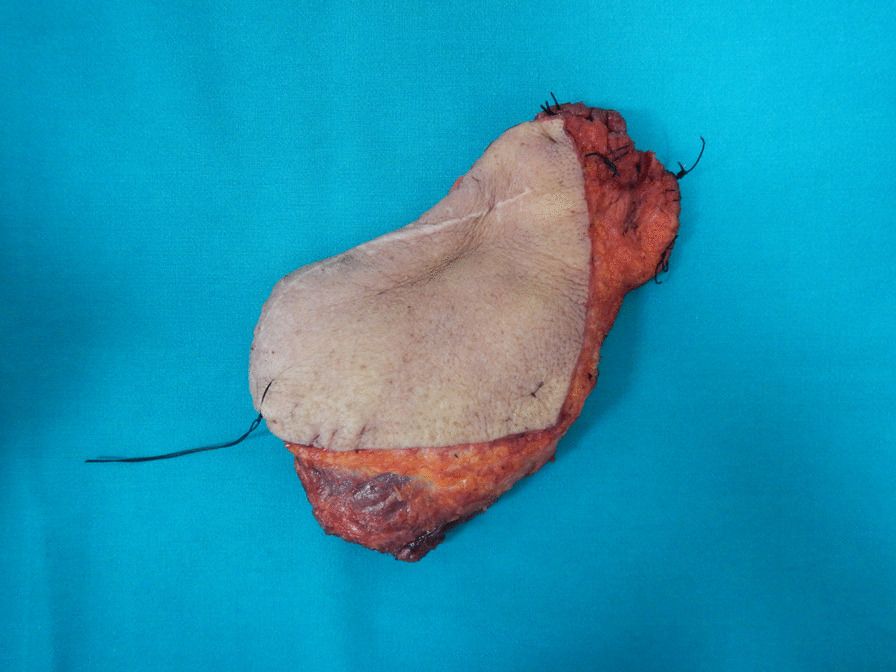
Surgical specimen measuring 17 cm × 11 cm × 4 cm

In June 2018, a wide surgical resection including the left trapezium muscle, superficial portion of the neck splenius muscle, and levator scapulae muscle was performed with a planned macroscopic margin of a minimum of 3 cm. The defect was reconstructed by a contralateral trapezius myocutaneous flap. The size of the excised specimen was 17 cm × 11 cm × 4 cm (Fig. 3).

Postoperative histological examination was negative for viable tumor cells, showing only a fibro-productive process with chronic inflammation and regressive aspects. The patient had a complete response to preoperative RT. After MTB discussion, no further adjuvant treatments were provided.

After surgery, the patient showed a mild reduction in free movement of the neck in all directions and movement of the shoulder (abduction: 30°; elevation: 30°; minimal possibility of intra- and extra-rotation); muscle strength evaluation was negatively influenced by pain (numeric rating scale > 5). He had a mild disability involving some daily activities such as dressing, hygiene, rising, and carrying.

The patient had a regular postoperative course and was discharged on postoperative day 4, without systemic or local complications.

No relapse occurred in the 24-month postoperative follow-up, and complete recovery of performance in activities of daily living was observed.

## Discussion

MFS is a subtype of STS showing higher risk of local recurrence when compared to other STS types, with a reported recurrence rate of about 17–50% [8]. The rate of metastasis can be as high as 20–35% in intermediate- and high-grade MFS, and usually occurs in the lungs and bones. Distant metastasis is rarely seen in low-grade MFS. The 5-year overall survival rate for MFS is between 61 and 77% [9].

Head and neck MFS is extremely rare, and presentation in these challenging anatomical regions is a potential source of misdiagnosis and inappropriate surgery. For this reason, management of these patients in SRCs is strongly recommended.

According to ESMO-EURACAN Clinical Practice Guidelines [6], RT and wide surgical excision are recommended in the case of recurrent, deep, and large masses, especially in patients with a history of positive margins after inappropriate surgical excision, as in our case.

Extensive surgical resection, as the best chance to achieve negative margins, is related to long-term local control. Kikuta *et al*. retrospectively analyzed 30 patients with recurrent MFS. They described a 5-year recurrence-free survival rate of 9.8 and 62.3% for those with a positive histological margin and those with a negative margin, respectively. These data indicate that a positive margin is a significant predictor of poor prognosis [10].

The real impact of RT in STS, and specifically in MFS, is uncertain. The number of cases reported in the literature is so limited that it is not possible to clearly evaluate the usefulness and the best timing for RT in this specific histology. Nevertheless, Le Grange *et al*. described, in a retrospective review, a reduction of STS mass volume [the most common histological subtypes analyzed were myxoid liposarcoma (32%) and MFS (16%)] in 80% of cases (mainly myxoid liposarcoma) after preoperative RT [11].

In a randomized trial involving multiple subtypes of STS, it was found that RT helped reduce the recurrence rate of sarcoma. However, subgroup analysis of the different histological types was not performed. In the surgery-alone cohort, the incidence of local recurrence during the follow-up period (median follow-up period was 19.7 years) was 4%, compared to 0% in those who also received RT [12].

In a large, multicenter retrospective series, Callegaro *et al*. found that radiation therapy could reduce the risk of local recurrence and improve survival, especially among patients affected by MFS [7]. However, Bismarck *et al*., and more recently Teurneau *et al*., did not confirm the positive effect of RT on local control in patients affected by MFS [8, 13].

In this case report, due to the challenging position of the mass, our MTB decided to administer RT in a neoadjuvant setting in order to reduce the irradiation field (and thus the radiation exposure of the joints of the neck and shoulder) and reduce long-term morbidity. A secondary advantage of neoadjuvant RT is to decrease the tumor size and so reduce the extent of the surgery. In fact, in a delicate anatomical region such as the head or the neck, an extensive surgical procedure is not always feasible, because it may require the mutilating sacrifice of major motor function to obtain safety margins. For these reasons, the involvement of a dedicated multidisciplinary team, as in our center, is essential, even including a plastic surgeon, which allows for optimal reconstruction surgery, and a physiotherapist to provide a dedicated motor recovery program.

The peculiarity of this clinical case was that we obtained an uncommon radiological response after RT, with a dimensional decrease of the neoplasm of about 50%. Only Cante *et al*. have described a case of a maxillary sinus MFS with a complete radiological response after combined treatment with RT and chemotherapy (CHT) [14].

However, even more surprising was the complete pathological response to preoperative RT that, to our knowledge, is the first described in literature.

## Conclusions

In MFS, as in all STS, a multidisciplinary assessment, one of the peculiarities of SRCs, is crucial for guaranteeing the best oncological and functional outcome.

RT seems to promise an optimal chance for local control in MFS patients.

Further studies are needed to confirm whether the observed efficacy of combined treatments for MFS results in a prolonged time of disease-free survival and overall survival.

## Data Availability

Not applicable.

## Abbreviations

MFS: Myxofibrosarcoma
STS: Soft tissue sarcoma
SRC: Sarcoma referral center
MRI: Magnetic resonance imaging
PET/CT: Positron-emission tomography/computed tomography
RT: Radiotherapy
CHT: Chemotherapy

## References

[1] Dell’Aversana Orabona G, Iaconetta G, *et al*. Head and neck myxofibrosarcoma: a case report and review of the literature. J Med Case Rep. 2014;8:468. 10.1186/1752-1947-8-468 PMC4320505 25547541

[2] Roland CL, Wang WL, *et al*. Myxofirbosarcoma. Surg Oncol Clin N Am. 2016;25(4):775–88. 10.1016/j.soc.2016.05.008 27591498

[3] Merk C, Angervall L, *et al*. Myxofibrosarcoma. A malignant soft tissue tumor of fibroblastic-histiocytic origin. A clinicopathologic and prognostic study of 110 cases using multivariate analysis. Acta Pathol Microbiol Immunol Scand Suppl. 1983;282:1–40. 6444190

[4] Mansoor A, White CR Jr. Myxofibrosarcoma presenting in the skin: clinicopathological features and differential diagnosis with cutaneous myxoid neoplasm. Am J Dermatophatol. 2003;25(4):2–11. 10.1097/00000372-200308000-00001 12876484

[5] Tjarks BJ, Ko JS, Billings SD. Myxofibrosarcoma of unusual sites. J Cutan Pathol. 2018;45(2):104–10. 10.1111/cup.13063. 29065228

[6] Casali PG, Abecassis N, *et al*. Soft tissue and visceral sarcomas: ESMO–EURACAN Clinical Practice Guidelines for diagnosis, treatment and follow-up. Ann Oncol. 2018;29:iv51–67. 10.1093/annonc/mdy096 29846498

[7] Callegaro D, Miceli R, Bonvalot S, *et al*. Impact of perioperative chemotherapy and radiotherapy in patients with primary extremity soft tissue sarcoma: retrospective analysis across major histological subtypes and major reference centres. Eur J Cancer. 2018;105:19–27. 10.1016/j.ejca.2018.09.028 30384013

[8] Teurneau H, Engellau J, *et al*. High recurrence rate of myxofibrosarcoma: the effect of radiotherapy is not clear. Hindawi Sarcoma. 2019;2019:8. 10.1155/2019/8517371 PMC6791216 31662702

[9] Sanfilippo R, Miceli R, *et al*. Myxofibrosarcoma: prognostic factors and survival in a series of patients treated at a single institution. Ann Surg Oncol. 2011;18(3):720–5. 10.1245/s10434-010-1341-4. 20878245

[10] Kikuta K, Nakayama R, Yoshida A, Sasaki A, Kameyama K, Chuman H, Kawai A, Nakamura M, Matsumoto M, Morioka H. A histological positive margin after surgery is correlated with high local re-recurrence rate in patients with recurrent myxofibrosarcoma. Jpn J Clin Oncol. 2017;47(4):334–41. 10.1093/jjco/hyw199. 28064204

[11] Le Grange F, Cassoni AM, *et al*. Tumour volume changes following pre-operative radiotherapy in borderline resectable limb and trunk soft tissue sarcoma. Eur J Surg Oncol. 2014;40(4):394–401. 10.1016/j.ejso.2014.01.011 24534361

[12] Beane JD, Yang JC, White D, *et al*. Efficacy of adjuvant radiation therapy in the treatment of soft tissue sarcoma of the extremity: 20-year follow-up of a randomized prospective trial. Ann Surg Oncol. 2014;21:2484–9. 10.1245/s10434-014-3732-4. PMC6293463 24756814

[13] Bismarck O, Jean-Claud BS, *et al*. Predictors of local recurrence in patients with myxofibrosarcoma. Am J Clin Oncol. 2017;00:000–000. 10.1097/COC.0000000000000382 28640064

[14] Cante D, Franco P, *et al*. Combined chemoradiation for head and neck region myxofibrosarcoma of the maxillary sinus. Tumori. 2013;99:e80–3. 10.1177/030089161309900235 23748836

[15] Group SS. SSG XXIV, recommendations for radiotherapy in bone- and soft tissue sarcoma. 2015

[16] Haas RL, Delaney TF, O’Sullivan B, *et al*. Radiotherapy for management of extremity soft tissue sarcomas: why, when, and where? Int J Radiat Oncol Biol Phys. 2012;84:572–80. 10.1016/j.ijrobp.2012.01.062 22520481

[17] Sampath S, Timothy MD, Schultheiss E, *et al*. Preoperative versus postoperative radiotherapy in soft-tissue sarcoma: multi-institutional analysis of 821 patients. Int J Radiat Oncol Biol Phys. 2011;81(2):498–505. 10.1016/j.ijrobp.2010.06.034 20888702

[18] Yang JC, Chang AE, Baker AR, *et al*. Randomized prospective study of the benefit of adjuvant radiation therapy in the treatment of soft tissue sarcomas of the extremity. J Clin Oncol. 1998;16:197–203. 10.1200/JCO.1998.16.1.197. 9440743

[19] Li Z, Liu X, Zhang Q, Zhang J, Huang M, Liu S. Myxofibrosarcoma of the mandible: a case report and review of the literature. BMC Oral Health. 2020;20(1):113. 10.1186/s12903-020-01094-7. PMC7164169 32299394

